# Psychological aspects of the development of individual programs to prevent periodontal diseases

**DOI:** 10.1186/1878-5085-5-S1-A112

**Published:** 2014-02-11

**Authors:** Liudmila Orekhova, Elena Isaeva, Vadim Tachalov, Ekaterina Loboda

**Affiliations:** 1 Therapeutic Dentistry Department of SSMU, St. Petersburg, Russia City Periodonrology Center "PAKS", St. Petersburg, Russia

## Introduction

Individual approach to treating patients as well as to preventing the development and progression of periodontal disease is best accomplished in the context of due consideration of individual’s psychological features. The aim of this study is to create efficient programs for periodontal disease prevention taking into consideration patient’s individual psychological status.

## Materials and methods

The study enrolled 140 individuals who consented to the study requirements. The dental examination included assessment of dental status, oral hygiene index and periodontal index as well as completion of a questionnaire regarding patient’s reasons for the dental visit. The psychodiagnostic tests that were included in the study design were the Interpersonal Diagnosis of Personality (T.Leary, 1957), Big Five Personality Test (L. Goldberg et al., 1985), Rotter's Locus of Control Scale (G.Potter, 1966) and, Test anxiety (C.D. Spielberger, 1995).

## Results

The data suggest reliable relation (p<0.05) between personality type (style interactions of personality) and patient’s attitude to personal dental health and prophylaxis. Those who follow dentist’s recommendations are emotionally stable, extroverts with low levels of anxiety and prone to competitive style of interactions with the highest rates of their cooperation (readiness to comply with the recommendations) (m=6,0±1,4; p<0,05) as well as the psychological resources (learning capability, openness to new experiences and readiness to perceive information).

Patients with responsible altruistic and aggressive-independent style of interactions are the second most compliant subjects with the hygienic recommendations and display highest rates of cooperation and psychological resources; this category of patients frequently visit dental professionals.

On the other hand, those who rarely visit a dentist (unless suffering from an acute episode of pain/discomfort) are prone to be stressed and withdrawn (or rebellious) and possess the lowest self-organization and self-control rates (m=1,6±1,3 и m=1,0±0,5) as well as psychological resources (m=3,0±0,1). Finally, patients who are dependent on others for their daily affairs rarely visit their dentist. These individuals are prone to follow their habits and lack self-discipline and are least cooperative (m=3, 2±1, 8).

## Conclusions

Psychological status of patients is a key determinant of their desire to see dental care and adhere to recommendations to maintain oral health. These aspects should be carefully considered by health care professionals as they ender care to patients.

